# Prevalence of Homologous Recombination Repair Mutations and Association with Clinical Outcomes in Patients with Solid Tumors: A Study Using the AACR Project GENIE Dataset

**DOI:** 10.3390/cancers17040577

**Published:** 2025-02-08

**Authors:** Changxia Shao, Heng Zhou, Cai Chen, Elisha J. Dettman, Yixin Ren, Razvan Cristescu, Alexander Gozman, Fan Jin

**Affiliations:** Merck & Co., Inc., Rahway, NJ 07065, USA; heng.zhou@merck.com (H.Z.); cai.chen@merck.com (C.C.); elisha.dettman@merck.com (E.J.D.); yixin.ren@merck.com (Y.R.); razvan_cristescu@merck.com (R.C.); alexander.gozman@merck.com (A.G.); fan.jin@merck.com (F.J.)

**Keywords:** homologous recombination repair mutation, HRRm, *BRCA1* and *BRCA2* mutation, BRCAm, prognosis, solid tumors, breast cancer, prostate cancer, pancreatic cancer

## Abstract

Background/Objectives: Mutations in *BRCA1* and/or *BRCA2* (BRCAm) and other homologous recombination repair genes (HRRm) are associated with several cancers. We evaluated the prevalence and association with overall survival (OS) of somatic BRCAm and HRRm among patients with advanced solid tumors. Methods: We used deidentified data from the AACR GENIE Biopharma Collaborative dataset derived from patients with tumors genotyped using next-generation sequencing between 1 January 2014 and 31 December 2017. Eligible patients were aged ≥18 years diagnosed with non-small-cell lung, colorectal, breast, bladder, prostate, or pancreatic cancer, with documented BRCA/HRR somatic mutation status. The primary analysis was OS (censored at the start of poly[ADP ribose] polymerase inhibitors [PARPi]/immunotherapy) after initiation of second-line therapy since most patients had sequencing after first-line therapy. Results: Among eligible patients, 242/7022 (3.4%) had BRCAm and 477/5474 (8.7%) had HRRm. Adjusted OS HRs (95% CI) for the primary analysis (using the initiation of second-line therapy as index date) were 0.79 (0.61–1.03) with/without BRCAm (n = 116/n = 3394) and 0.83 (0.69–0.99) with/without HRRm (n = 247/n = 2656); in sensitivity analysis of patients with stage IV disease, HRs were 0.97 (0.68–1.38) with/without BRCAm (n = 58/n = 1847) and 0.92 (0.73–1.18) with/without HRRm (n = 132/n = 1488). Conclusions: Overall, BRCAm and HRRm did not show a strong association with OS, with a trend toward longer OS among patients receiving standard second-line therapies excluding PARPi/immunotherapy.

## Simple Summary

This study looked at mutations in BRCA genes and others referred to as homologous recombination repair (HRR) genes. Mutations in these genes are linked to problems in the DNA repair process, which can lead to cancer. Data from patients in a real-world setting were used to see how often these mutations occur and how they affect survival rates. We found that 3.4% of patients with cancer had tumors with mutations in BRCA genes, and 8.7% had tumors with mutations in HRR genes. We also found that among patients who had received at least two lines of standard treatment for advanced cancer, there was a trend toward longer survival in patients with these mutations compared to those without. This information is important for understanding the role of these genetic changes in different cancers and improving cancer treatment.

## 1. Introduction

The DNA damage response comprises a set of cellular pathways that play a critical role in preserving genomic integrity and stability [1,2]. Defects in these repair mechanisms caused by homologous recombination repair gene mutations (HRRm) may predispose cells toward the development and progression of cancer [3,4,5,6]. Well-known examples include mutations in *BRCA1* and/or *BRCA2* (BRCAm), which increase the risk for breast and ovarian cancers [4,7]. BRCAm and other HRRm have also been found at varying frequencies in other tumor types, particularly in prostate and also in pancreatic cancers [7,8].

There is some evidence that these genetic mutations may be predictive of responses to specific treatments. For example, platinum chemotherapy has been associated with better clinical outcomes compared with other types of chemotherapy in BRCAm breast and pancreatic cancers [9,10]. Numerous clinical trials have also demonstrated the efficacy of poly (ADP ribose) polymerase inhibitors (PARPi) in BRCAm breast, ovarian, pancreatic, and prostate cancers [11,12,13,14,15,16] and in HRRm prostate cancer [17]. Additionally, studies evaluating the combination of PARPi and immunotherapy have shown associations between mutations in DNA damage repair genes, including BRCAm, and better efficacy across multiple tumor types [18,19,20]. Clinical trials are evaluating the combination of olaparib (PARPi) plus durvalumab (anti-programmed cell death ligand 1 antibody) and tremelimumab (anti–cytotoxic T-lymphocyte-associated antigen 4) in patients with HRRm solid tumors [21] and olaparib plus pembrolizumab (anti-programmed cell death protein 1 antibody) in patients with BRCAm, HRRm, and/or homologous recombination deficiency–positive solid tumors [22].

Because HRR biomarkers have been associated with clinical outcomes in patients with solid tumors receiving PARPi and immunotherapy, understanding whether these biomarkers have a prognostic impact on clinical outcomes is critical for interpreting results from such studies. Large, real-world studies in patients not receiving these treatments are needed to evaluate the prognostic value of BRCAm and HRRm across solid tumors, as limited existing data have demonstrated contrasting results. Two studies in patients receiving standard therapies for ovarian cancer have demonstrated a positive association between BRCAm and overall survival (OS) [23,24], whereas another study found no association between HRRm and OS in patients receiving standard treatments for high-grade serous ovarian cancers [25]. A prospective cohort study in patients receiving routine care for early-onset breast cancer also found no association between BRCAm status and OS [26].

The American Association for Cancer Research (AACR) Genomics Evidence Neoplasia Information Exchange (GENIE) registry harmoniously combines and links next-generation sequencing data with clinical outcomes data for patients with cancer receiving treatment at 1 of 19 participating academic centers. The GENIE Biopharma Collaborative (BPC) is a data-sharing effort across 10 biopharmaceutical companies to collectively gather deidentified comprehensive clinical and genomic data from patients treated at participating institutions [27]. This dataset provides a unique opportunity to evaluate the prevalence of BRCAm and HRRm across tumor types and to assess the prognostic value of these biomarkers in patients receiving standard treatments other than PARPi or immunotherapy.

## 2. Materials and Methods

### 2.1. Study Design and Patients

This retrospective observational cohort study used deidentified data from patients with solid tumors included in the AACR GENIE BPC phase 1 dataset. The phase 1 dataset includes information on prior cancer treatments, tumor pathology, and clinical outcomes added during the first 2 years of the GENIE BPC, which were already linked with genomic profiles of nearly 8000 patients with one of six solid tumor types: bladder, breast, colorectal (CRC), non–small-cell lung (NSCLC), pancreatic, or prostate cancer [27]. Data for the present study were derived from patients whose tumors were genotyped between 1 January 2014 and 31 December 2017 at one of four academic centers: Memorial Sloan Kettering Cancer Center, USA (MSK); Dana–Farber Cancer Institute, USA (DFCI); Vanderbilt–Ingram Cancer Center, USA (VICC); or Princess Margaret Cancer Centre, University Health Network, Canada (UHN). Clinical data were collected from electronic health records, with data collection managed using a common, secure web-based Research Electronic Data Capture (REDCap) database following a harmonized data collection guide [28]. OS data were derived from institutional electronic health record data, which were linked to the National Death Index.

Eligible patients had a pathologist-confirmed diagnosis of one of the six solid tumor types included in the phase 1 database, were aged ≥18 years old at the time of tumor genomic sequencing, and had documented *BRCA* or HRR mutation status. Patients without available information on their *BRCA* or HRR mutation status or who were diagnosed with a tumor of not otherwise specified histology were excluded from the study. The GENIE BPC database is deidentified; therefore, this study was exempt from review by an institutional review board and included a waiver of informed consent.

### 2.2. Biomarker Assessment

This study evaluated the prevalence of BRCAm (i.e., mutations in the *BRCA1* and/or *BRCA2* genes) and HRRm, which were defined according to principles from the American College of Medical Genetics and Genomics standards and guidelines for interpreting sequence variants [29]. HRRm included mutations in any of the following 15 genes: *BRCA1*, *BRCA2*, *ATM*, *BARD1*, *BRIP1*, *CDK12*, *CHEK1*, *CHEK2*, *FANCL*, *PALB2*, *PPP2R2A*, *RAD51B*, *RAD51C*, *RAD51D*, and *RAD54L*. Gene panels with fewer than 10 HRR genes were excluded from HRRm-related analyses. Mutation status was evaluated in tumor tissue and reported as a dichotomous variable (mutated [BRCAm or HRRm] vs. wild-type [BRCAwt or HRRwt]). Mutations of somatic origin were evaluated; germline mutation data were not available from the GENIE database due to patient privacy [28].

### 2.3. Statistical Analysis

The primary objectives of this study were to estimate the prevalence of BRCAm and HRRm across tumor types in a real-world setting and to evaluate the association between these biomarkers and OS among patients receiving routine clinical care for one of the six included tumor types. OS analyses were performed from the following index dates, based on available data: initiation of first-line, second-line, and third-line therapy. The primary analysis was based on the start of second-line therapy as the index date since most patients had tumor genomic sequencing after initiation of first-line therapy. Exploratory analyses were also performed to evaluate the association between the HRR biomarkers and OS by individual tumor type. Categorical variables (e.g., demographics and baseline disease characteristics) were described according to biomarker status. Mean and median follow-up times were calculated based on numerical values.

The line of therapy was derived from the GENIE dataset and defined based on the following general rules: the line of therapy started from the first antineoplastic agent received after metastatic diagnosis (if initial diagnosis of stage IV disease) or after the first documented disease progression (per imaging or physician report if initial diagnosis of stage I–III disease); any treatment regimen received within 28 days of initiation of a line of therapy was considered part of the treatment regimen in that line of therapy; and discontinuation of a line of therapy was defined as either a treatment gap of ≥120 days, the addition of a new antineoplastic agent ≥ 28 days after starting the initial agents in the same line, or the end of cohort follow-up or last known contact, whichever was earliest.

Patient electronic health records were curated from the date of diagnosis to death for deceased patients; patients who remained alive were censored at their last known contact or end of cohort follow-up. OS was defined as the time from the index date to the date of death due to any cause or the censor date. Patients who initiated a PARPi (i.e., olaparib, niraparib, rucaparib, or talazoparib) or an immune checkpoint inhibitor (i.e., atezolizumab, avelumab, cemiplimab, durvalumab, ipilimumab, nivolumab, or pembrolizumab) or died before the index date or sequencing report date were excluded from the OS analysis, for delay entry adjustment. Patients who received one of these agents after the index date were censored at the time of initiating the agent. Patients included in the OS analysis otherwise received routine clinical care according to the line of therapy, including chemotherapy or targeted therapies directed at specific mutations (other than PARPi). Median OS and 95% CIs were estimated using the Kaplan–Meier product-limit method; landmark OS analyses were also performed to estimate the percentage of patients who remained alive at least 6 months, 1 year, and 2 years after the index date. The relationship between biomarker status and OS was assessed using Cox proportional hazards modeling, adjusted for age, gender, tumor type, and stage (for ‘all stages’ analysis). Left truncation was used for OS analysis to account for the delayed entry of genomic sequencing that occurred after the start of the index date for survival estimation. To better understand the prognostic effects of BRCA and HRR biomarkers in advanced solid tumors and support the interpretation of clinical studies of novel treatment approaches in this setting, a sensitivity analysis was conducted in the subgroup of patients with stage IV disease. The relationship between OS and biomarker status using initiation of first-line therapy and third-line therapy as index dates was also assessed in sensitivity analyses.

All statistical analyses were prespecified, with no adjustment for multiple comparisons. The analyses were performed between January 2023 and April 2024 using R statistical software version 4.1.0 (R Foundation, Vienna, Austria).

## 3. Results

### 3.1. Patient Population

**Figure 1:**
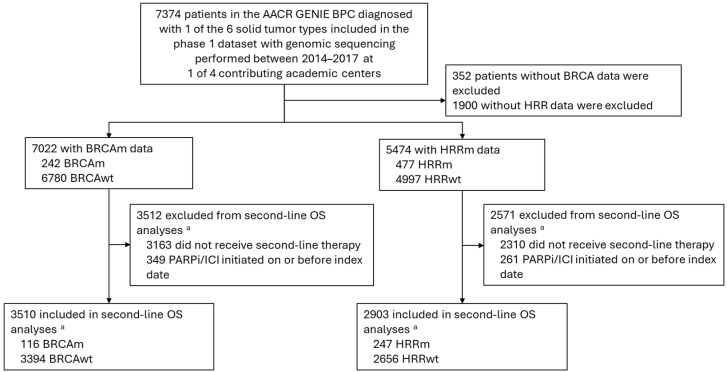
Study flow diagram. AACR GENIE BPC, American Association for Cancer Research Genomics Evidence Neoplasia Information Exchange Biopharma Collaborative; BRCA, *BRCA1* and/or *BRCA2*; BRCAm, BRCA mutation; BRCAwt, BRCA wild-type; HRRm, homologous recombination repair mutation; HRRwt, homologous recombination repair wild-type; ICI, immune checkpoint inhibitor; OS, overall survival; PARPi, poly (ADP ribose) polymerase inhibitor. ^a^ Primary analysis.

The study included 7022 eligible patients with any of the six specified tumor types (Figure 1). In the overall cohort, the mean age was 58.5 years old (SD, 12.8 years old), and 3372 patients (48.0%) were women. Demographics and baseline disease characteristics were largely consistent across patient groups with available data on their BRCA (n = 7022) and HRR (n = 5474) status and between patient groups with positive or negative biomarker status (Table 1). In the BRCA and HRR cohorts, 38.8% and 41.2% of patients, respectively, had stage IV disease at initial diagnosis. The proportions of patients with each tumor type were generally consistent within and between the BRCA and HRR cohorts, with bladder cancer being the least common tumor type in both cohorts (Table 1). The median duration of follow-up from initiation of second-line therapy was 23.2 months (interquartile range, 9.8–45.3 months) in the BRCA cohort and 22.7 months (interquartile range, 9.5–44.6 months) in the HRR cohort (Table 1). For both BRCA and HRR, median follow-up was consistent between the biomarker-positive and biomarker-negative groups (Table 1).

### 3.2. Treatment Patterns

Among patients who initiated second-line therapy (BRCA cohort [n = 3859]; HRR cohort [n = 3164]), most received chemotherapy (BRCA cohort, 59.4%; HRR cohort, 60.3%) or targeted therapy other than PARPi (BRCA cohort, 32.3%; HRR cohort, 31.4%); a minority of patients (both BRCA cohort and HRR cohort, 8.3%) received PARPi/immunotherapy and were censored from the OS analysis (Table A1).

### 3.3. Prevalence of Somatic BRCA and HRR Biomarkers

Across tumor types, BRCAm was identified in 242 of 7022 patients (3.4%) and HRRm in 477 of 5474 patients (8.7%), with variable prevalence by tumor type (Table 2). BRCAm was most prevalent among patients with CRC (65 of 1430 patients [4.5%]), and the lowest prevalence was observed among patients with NSCLC (27 of 1623 patients [1.7%]). HRRm was most prevalent among patients with prostate cancer (104 of 831 patients [12.5%]), and the prevalence was lowest among patients with breast cancer (49 of 855 patients [5.7%]). Among patients with stage IV disease at initial diagnosis, BRCAm was identified in 82 of 2724 patients (3.0%), and HRRm was identified in 185 of 2255 patients (8.2%; Table 1 and Table A2).

### 3.4. Association of BRCAm and HRRm with Overall Survival

For the primary analysis of OS among patients who initiated second-line therapy and did not receive PARPi or immunotherapy, 3510 patients had available data on BRCAm status (BRCAm, n = 116; BRCA wild-type [BRCAwt], n = 3394; Table 3). From the start of second-line therapy, median OS was 22.4 months (95% CI, 16.4–31.3 months) in patients with BRCAm versus 17.0 months (95% CI, 15.9–18.1 months) in those without BRCAm (Table 3); adjusted hazard ratio (HR) was 0.79 (95% CI, 0.61–1.03) and estimated 2-year OS rates were 45% and 39% in the BRCAm and BRCAwt groups, respectively. In a sensitivity analysis in the subgroup of patients with stage IV tumors that was also indexed at the start of second-line therapy, median OS for the 58 patients with stage IV BRCAm tumors was 21.5 months (95% CI, 17.3–35.3 months) compared with 16.7 months (95% CI, 14.8–18.6 months) for the 1847 patients with stage IV BRCAwt tumors (Table 3); adjusted HR was 0.97 (95% CI, 0.68–1.38) and estimated 2-year survival rates were 46% and 39% in the BRCAm and BRCAwt groups, respectively. OS was further assessed from the start of second-line therapy by tumor type (Table A3). For CRC, which had the highest prevalence of BRCAm, the adjusted HR for OS for the BRCAm versus BRCAwt groups was 0.62 (95% CI, 0.25–1.52; Table A3). Sensitivity analyses using the start of first-line and the start of third-line therapy as index dates resulted in adjusted HRs for OS of 0.79 (95% CI, 0.62–1.02) and 0.90 (95% CI, 0.68–1.19), respectively, for the BRCAm versus BRCAwt groups (Table 3).

The mutation status for HRR was available from 2903 patients for the primary analysis of OS (HRRm, n = 247; HRR wild-type [HRRwt], n = 2656; Table 3). From the start of second-line therapy, median OS in patients with versus without HRRm was 22.4 months (95% CI, 17.4–25.1 months) versus 16.6 months (95% CI, 15.2–17.9 months; Table 3); adjusted HR was 0.83 (95% CI, 0.69–0.99) and estimated 2-year OS rates were 44% and 39% in patients with versus without HRRm, respectively. In a sensitivity analysis in the subgroup of patients with stage IV tumors that was also indexed at the start of second-line therapy, the median OS for the 132 patients with stage IV HRRm tumors was 19.6 months (95% CI, 16.9–32.0 months) compared with 15.9 months (95% CI, 14.1–18.0 months) for the 1488 patients with stage IV HRRwt tumors (Table 3); adjusted HR was 0.92 (95% CI, 0.73–1.18) and estimated 2-year survival rates were 44% and 38% in the HRRm and HRRwt groups, respectively. Additionally, for prostate cancer, which had the highest prevalence of HRRm, adjusted HR for OS for the HRRm versus HRRwt groups was 0.92 (95% CI, 0.63–1.34) (Table A3). Sensitivity analyses using the start of first-line and the start of third-line therapy as index dates resulted in adjusted HRs for OS of 0.80 (95% CI, 0.67–0.95) and 0.90 (95% CI, 0.74–1.10), respectively, for the HRRm versus HRRwt groups (Table 3).

## 4. Discussion

This large, retrospective, observational cohort study utilizing comprehensive, curated clinical and outcomes patient-level data from the GENIE BPC database fills a need for real-world evidence on the prevalence and prognostic impact of BRCAm and HRRm across several solid tumor types, including bladder cancer, breast cancer, CRC, NSCLC, pancreatic cancer, and prostate cancer. We found that the prevalence of somatic BRCAm and HRRm across these six tumor types was 3.4% and 8.7%, respectively. The frequency of these biomarkers varied by tumor type, ranging from 1.7% to 4.5% for BRCAm, which was most common among patients with CRC, and from 5.7% to 12.5% for HRRm, which was most common among patients with prostate cancer. Notably, because data for germline mutations were not available from the GENIE BPC database, the prevalence rates we observed are lower than those reported in previous studies that included data for both somatic and germline mutations. A prior study using data from the US Flatiron Health-Foundation Medicine clinico-genomic database (FH-FMI CGDB), where both somatic and genomic mutations were included, found overall prevalence rates of 4.7% for BRCAm and 13.6% for HRRm across 15 solid tumor types, with rates for the six tumor types included in the present study ranging from 3.1% to 9.3% for BRCAm and 10.5% to 26.0% for HRRm [30]. Additionally, a previous study that assessed the prevalence of these biomarkers in prostate cancer and used data from both FH-FMI CGDB and the GENIE database found prevalence rates of 9.2% and 24.6% for BRCAm and HRRm, respectively, when considering both somatic and germline mutations, and lower rates of 3.7% and 11.0% when considering only somatic mutations [31]. Importantly, the latter prevalence rates compare closely to those we observed for prostate cancer in our study (3.9% for BRCAm and 12.5% for HRRm).

In our primary analysis of OS across tumor types, which used the start of second-line therapy as the index date since most patients had tumor genomic sequencing after initiating first-line therapy, we found that BRCAm and HRRm were not strongly associated with OS among patients not receiving PARPi or immunotherapy. Results from sensitivity analyses using the start of first-line and the start of third-line therapy as index dates were consistent with these findings. Analyses in patients with stage IV disease at initial diagnosis were also consistent with these results. In patients with tumor types that had the highest prevalence of each HRR biomarker (i.e., CRC for BRCAm and prostate cancer for HRRm), we also found no association between the respective biomarkers and OS in these tumor types. Due to small sample sizes, interpretation of the association between HRR biomarkers and OS in each tumor subgroup is limited; however, HRRm did not appear to be strongly associated with OS in patients with any of the six tumor types included.

Our results from the sensitivity analysis among patients who had stage IV tumors are consistent with those from a previous study based on data from the FH-FMI CGDB [32]. In that study, the primary analysis using the start of second-line therapy as the index date and including a cohort of patients with 1 of 15 tumor types at advanced or metastatic stage receiving standard therapy excluding PARPi and immunotherapy demonstrated no association between OS and either BRCAm (adjusted HR, 0.83 [95% CI, 0.60–1.17]) or HRRm (adjusted HR, 0.95 [95% CI, 0.79–1.14]). Estimated 2-year survival rates across the large number of tumor types in the FH-FMI CGDB study were 33% versus 36% among patients with versus without BRCAm and 33% versus 35% with versus without HRRm, which were comparable to the rates observed in the present study (with vs. without BRCAm, 45% vs. 39%; with vs. without HRRm, 44% vs. 39%). Additionally, our overall findings were generally consistent with results from a prior systematic review and meta-analysis that evaluated the prognostic impact of BRCAm and HRRm on OS in patients with various solid tumors who received chemotherapy or targeted therapy, excluding PARPi [33]. That meta-analysis found no association between BRCAm and OS in patients with breast or pancreatic cancer (HRs for OS for BRCAm vs. BRCAwt, 1.02 [95% CI, 0.80–1.30] and 1.53 [95% CI, 0.76–3.06], respectively) or between HRRm and OS overall across the 5 included cancer types in the study (breast, bladder, pancreatic, ovarian, and prostate cancer; HR, 0.99 [95% CI, 0.71–1.38]). Notably, the POSH study in patients receiving standard therapy for early-onset breast cancer (N = 2733) found no significant difference in OS between patients with or without BRCAm tumors, including in an analysis adjusted for prognostic factors such as ethnicity and body mass index (HR, 0.96 [95% CI, 0.76–1.22]) [26].

The limitations of this study include that patient data were collected at four academic centers among patients with tumors that were sequenced between 1 January 2014 and 31 December 2017, and it may not be representative of the general population of patients treated for cancer across clinical settings. The sequencing criteria may introduce immortal bias. Although delay entry adjustment was applied, the method may not fully address the time lapse between molecular testing and diagnosis within clinico-genomic data [34]. Thus, the absolute values of OS for each subgroup should be interpreted with caution. Due to small sample sizes in individual tumor subgroups, the Cox model used for the OS analysis was not adjusted for prognostic factors that are specific to individual tumor types. Additionally, the observed prevalence of BRCAm and HRRm reflects somatic mutations only, which could underestimate the prevalence of BRCAm and HRRm. There was also no standardized point for tumor sample collection relative to diagnosis or treatment journey, nor were specific mutation definitions standardized across sequencing panels. However, biomarker data were derived from a large database of patient-level data in a real-world setting, and HRR biomarker status was determined using next-generation sequencing, which is commonly used in practice. Finally, the standard of care and clinical practice has evolved since the start of this study, including the use of PARPi and immunotherapy, which are now standard treatments for some types of cancer [35,36]. For example, PARPi are now standard therapies for breast, prostate, and pancreatic cancers with BRCAm [36,37,38]. Therefore, our methodology to censor patients at the time of initiating these treatments may have led to lower-than-expected event rates for OS and may have limited our assessment of the prognostic impact of BRCAm in these tumor types. However, since BRCAm and HRRm have been shown to predict treatment outcomes with PARPi and immunotherapy across several tumor types, including breast, pancreatic, and prostate cancer [12,13,14,15,16,17,18,19], it was necessary to exclude these therapies from our OS analysis in order to assess the prognostic effects of the HRR biomarkers.

## 5. Conclusions

Results from this large, retrospective cohort study using real-world data demonstrated variable prevalence of BRCAm and HRRm across tumor types, with the highest prevalence of somatic BRCAm in patients with CRC and the highest prevalence of somatic HRRm in patients with prostate cancer. Among patients receiving standard second-line therapies other than PARPi or immunotherapy, there was no strong association between OS and BRCAm or HRRm. These findings enrich the general understanding of the prevalence and prognostic effects of these HRR biomarkers and provide context to support the interpretation of results from studies evaluating the use of PARPi and immunotherapy in solid tumors.

## Figures and Tables

**Table 1:** Demographics and baseline disease characteristics in patients with solid tumors by HRR biomarker status.

	BRCA			HRR		
	BRCAm n = 242	BRCAwt n = 6780	Total n = 7022 ^a^	HRRm n = 477	HRRwt n = 4997	Total n = 5474 ^a^
Sex						
Female	108 (44.6)	3264 (48.1)	3372 (48.0)	187 (39.2)	2432 (48.7)	2619 (47.8)
Male	134 (55.4)	3516 (51.9)	3650 (52.0)	290 (60.8)	2565 (51.3)	2855 (52.2)
Age, median/mean (SD), y	60/57.1 (13.8)	60/58.6 (12.8)	60/58.5 (12.8)	61/59.6 (12.8)	59/58.4 (12.8)	60/58.5 (12.8)
<65 y	164 (67.8)	4313 (63.6)	4477 (63.8)	285 (59.7)	3203 (64.1)	3488 (63.7)
Race						
White	213 (88.0)	5723 (84.4)	5936 (84.5)	412 (86.4)	4120 (82.4)	4532 (82.8)
Black	12 (5.0)	346 (5.1)	358 (5.1)	20 (4.2)	286 (5.7)	306 (5.6)
Asian	8 (3.3)	315 (4.6)	323 (4.6)	17 (3.6)	259 (5.2)	276 (5.0)
Other/missing	9 (3.7)	396 (5.8)	405 (5.8)	28 (5.9)	332 (6.6)	360 (6.6)
Disease stage						
I–III	160 (66.1)	4126 (60.9)	4286 (61.0)	291 (61.0)	2918 (58.4)	3209 (58.6)
IV	82 (33.9)	2642 (39.0)	2724 (38.8)	185 (38.8)	2070 (41.4)	2255 (41.2)
0 or missing	0	12 (0.2)	12 (0.2)	1 (0.2)	9 (0.2)	10 (0.2)
Academic center						
DFCI	114 (47.1)	2767 (40.8)	2881 (41.0)	151 (31.7)	1182 (23.7)	1333 (24.4)
MSK	95 (39.3)	3384 (49.9)	3479 (49.5)	254 (53.2)	3225 (64.5)	3479 (63.6)
UHN	7 (2.9)	76 (1.1)	83 (1.2)	13 (2.7)	70 (1.4)	83 (1.5)
VICC	26 (10.7)	553 (8.2)	579 (8.2)	59 (12.4)	520 (10.4)	579 (10.6)
Tumor type						
Bladder	27 (11.2)	667 (9.8)	694 (9.9)	45 (9.4)	463 (9.3)	508 (9.3)
Breast	45 (18.6)	1084 (16.0)	1129 (16.1)	49 (10.3)	806 (16.1)	855 (15.6)
CRC	65 (26.9)	1365 (20.1)	1430 (20.4)	133 (27.9)	1016 (20.3)	1149 (21.0)
NSCLC	27 (11.2)	1596 (23.5)	1623 (23.1)	83 (17.4)	1137 (22.8)	1220 (22.3)
Pancreatic	36 (14.9)	1022 (15.1)	1058 (15.1)	63 (13.2)	848 (17.0)	911 (16.6)
Prostate	42 (17.4)	1046 (15.4)	1088 (15.5)	104 (21.8)	727 (14.5)	831 (15.2)
Follow-up from initial diagnosis, mo						
Mean (SD)	57.9 (47.9)	50.3 (44.7)	50.5 (44.8)	53.4 (43.8)	48.9 (45.1)	49.3 (45.0)
Median (IQR)	45.5 (27.9–70.0)	39.0 (21.2–62.8)	39.2 (21.4–63.2)	40.7 (26.8–64.1)	37.5 (20.5–58.9)	37.8 (21.2–59.5)
Follow-up from start of second-line therapy, mo						
Mean (SD)	40.8 (45.3)	33.0 (33.5)	33.2 (34.0)	34.9 (33.3)	22.3 (34.0)	32.7 (33.9)
Median (IQR)	27.0 (13.0–51.4)	23.0 (9.7–45.0)	23.2 (9.8–45.3)	25.9 (12.7–46.9)	22.3 (9.4–44.4)	22.7 (9.5–44.6)

Data are n (%) unless otherwise noted. BRCA, *BRCA1* and/or *BRCA2***;** BRCAm, BRCA mutation; BRCAwt, BRCA wild-type; DFCI, Dana–Farber Cancer Institute; CRC, colorectal cancer; HRR, homologous recombination repair; HRRm, homologous recombination repair mutation; HRRwt, homologous recombination repair wild-type; MSK, Memorial Sloan Kettering; NSCLC, non-small-cell lung cancer; UHN, University Health Network; VICC, Vanderbilt–Ingram Cancer Center. ^a^ Includes all patients with available data for the respective biomarker.

**Table 2:** Prevalence of BRCA and HRR mutations by tumor type.

	BRCAm			HRRm		
Tumor Type	N	n	Prevalence (95% CI), %	N	n	Prevalence (95% CI), %
Pan-tumor	7022	242	3.4 (3.0–3.9)	5474	477	8.7 (8.0–9.5)
Bladder cancer	694	27	3.9 (2.6–5.6)	508	45	8.9 (6.5–11.7)
Breast cancer	1129	45	4.0 (2.9–5.3)	855	49	5.7 (4.3–7.5)
Colorectal cancer	1430	65	4.5 (3.5–5.8)	1149	133	11.6 (9.8–13.6)
Non-small-cell lung cancer	1623	27	1.7 (1.1–2.4)	1220	83	6.8 (5.5–8.4)
Pancreatic cancer	1058	36	3.4 (2.4–4.7)	911	63	6.9 (5.4–8.8)
Prostate cancer	1088	42	3.9 (2.8–5.2)	831	104	12.5 (10.3–15.0)

BRCAm, *BRCA1* and/or *BRCA2* mutation; HRRm, homologous recombination repair mutation.

**Table 3:** OS analysis in patients with solid tumors by HRR biomarker status.

OS Index Date	Biomarker Status	N	Median OS (95% CI), mo	Unadjusted HR (95% CI)	Adjusted HR (95% CI)	Survival Rate, % (95% CI)		
						6-Month	**1-Year**	**2-Year**
**All disease stages**								
Start of second-line therapy ^a^	BRCAm	116	22.4 (16.4–31.3)	0.84 (0.65–1.09)	0.79 (0.61–1.03)	80 (70–91)	73 (62–85)	45 (34–59)
	BRCAwt	3394	17.0 (15.9–18.1)			77 (76–79)	59 (57–62)	39 (37–41)
	HRRm	247	22.4 (17.4–25.1)	0.83 (0.70–1.00)	0.83 (0.69–0.99)	83 (76–90)	68 (61–77)	44 (36–52)
	HRRwt	2656	16.6 (15.2–17.9)			77 (75–79)	59 (56–61)	39 (37–41)
Start of first-line therapy	BRCAm	138	27.8 (19.5–36.8)	0.86 (0.67–1.1)	0.79 (0.62–1.02)	86 (77–97)	78 (68–91)	56 (44–70)
	BRCAwt	4232	20.7 (19.5–22.0)			85 (83–87)	67 (65–69)	45 (43–47)
	HRRm	298	29.0 (25.0–36.8)	0.80 (0.67–0.95)	0.80 (0.67–0.95)	90 (85–96)	77 (70–85)	59 (51–67)
	HRRwt	3272	20.0 (18.8–21.6)			85 (83–87)	66 (64–68)	44 (42–46)
Start of third-line therapy	BRCAm	93	15.8 (11.8–25.5)	0.92 (0.70–1.21)	0.90 (0.68–1.19)	81 (72–92)	62 (50–76)	35 (25–51)
	BRCAwt	2674	14.9 (13.7–16.1)			74 (72–76)	55 (53–58)	35 (33–37)
	HRRm	197	16.1 (13.1–19.7)	0.89 (0.74–1.09)	0.90 (0.74–1.10)	76 (69–84)	63 (55–72)	38 (30–47)
	HRRwt	2058	14.9 (13.4–16.3)			74 (71–76)	55 (52–57)	35 (33–38)
**Stage IV disease**								
Start of second-line therapy	BRCAm	58	21.5 (17.3–35.3)	1.04 (0.74–1.47)	0.97 (0.68–1.38)	74 (60–90)	74 (60–90)	46 (32–66)
	BRCAwt	1847	16.7 (14.8–18.6)			76 (74–79)	58 (56–61)	39 (37–42)
	HRRm	132	19.6 (16.9–32.0)	0.92 (0.72–1.16)	0.92 (0.73–1.18)	82 (74–92)	68 (59–79)	44 (35–56)
	HRRwt	1488	15.9 (14.1–18.0)			75 (72–78)	57 (54–60)	38 (35–41)
Start of first-line therapy	BRCAm	67	28.9 (21.5–44.8)	0.95 (0.67–1.34)	0.80 (0.56–1.15)	85 (73–98)	79 (66–94)	62 (48–81)
	BRCAwt	2240	21.2 (19.7–23.0)			84 (82–87)	67 (64–70)	46 (43–48)
	HRRm	154	29.0 (23.3–43.5)	0.81 (0.64–1.03)	0.77 (0.61–0.98)	89 (81–97)	77 (68–87)	59 (50–71)
	HRRwt	1780	20.8 (19.3–22.8)			84 (82–87)	66 (63–69)	45 (42–48)
Start of third-line therapy	BRCAm	43	10.4 (7.9–17.7)	1.38 (0.96–1.97)	1.39 (0.96–1.99)	74 (60–91)	45 (30–66)	25 (14–47)
	BRCAwt	1350	15.0 (13.4–16.6)			75 (72–78)	55 (52–59)	36 (33–39)
	HRRm	100	15.0 (11.5–23.4)	0.99 (0.77–1.29)	1.01 (0.78–1.31)	75 (65–86)	58 (48–71)	36 (27–49)
	HRRwt	1077	14.4 (12.2–16.4)			74 (71–77)	54 (50–58)	35 (32–39)

BRCA, *BRCA1* and/or *BRCA2*; BRCAm, BRCA mutation; BRCAwt, BRCA wild-type; HRR, homologous recombination repair; HRRm, homologous recombination repair mutation; HRRwt, homologous recombination repair wild-type; OS, overall survival. ^a^ Primary analysis.

## Data Availability

AACR Project GENIE data are currently available via two mechanisms: 1. Synapse Platform (SageBionetworks): https://synapse.org/genie; 2. cBioPortal for Cancer Genomics (MSK): https://www.cbioportal.org/genie/.

## Appendix A

**Table A1:** Treatment patterns for each line of therapy by HRR biomarker status.

Treatment Category	BRCA			HRR		
	BRCAm	BRCAwt	Total	HRRm	HRRwt	Total
First-line regimen						
Total	155	4607	4762	332	3556	3888
PARPi	0 (0)	1 (0)	1 (0)	0 (0)	1 (0)	1 (0)
Immunotherapy	3 (1.9)	147 (3.2)	150 (3.2)	12 (3.6)	121 (3.4)	133 (3.4)
Other targeted therapy	32 (20.7)	946 (20.5)	978 (20.5)	56 (16.9)	693 (19.5)	749 (19.3)
Chemotherapy	120 (77.4)	3513 (76.3)	3633 (76.3)	264 (79.5)	2741 (77.1)	3005 (77.3)
Alkylating agents	64 (41.3)	2039 (44.3)	2103 (44.2)	139 (41.9)	1540 (43.3)	1679 (43.2)
Platinum-based	51 (32.9)	1713 (37.2)	1764 (37.0)	123 (37.1)	1315 (37.0)	1438 (37.0)
Non-platinum-based	13 (8.4)	326 (7.1)	339 (7.1)	16 (4.8)	225 (6.3)	241 (6.2)
Other	56 (36.1)	1474 (32.0)	1530 (32.1)	125 (37.7)	1201 (33.8)	1326 (34.1)
Second-line regimen						
Total	131	3728	3859	273	2891	3164
PARPi	3 (2.3)	2 (0.1)	5 (0.1)	2 (0.7)	2 (0.1)	4 (0.1)
Immunotherapy	15 (11.5)	300 (8.1)	315 (8.2)	33 (12.1)	227 (7.9)	260 (8.2)
Other targeted therapy	41 (31.3)	1206 (32.4)	1247 (32.3)	82 (30.0)	910 (31.5)	992 (31.4)
Chemotherapy	72 (55.0)	2220 (59.6)	2292 (59.4)	156 (57.1)	1752 (60.6)	1908 (60.3)
Alkylating agents	16 (12.2)	505 (13.6)	521 (13.5)	29 (10.6)	372 (12.9)	401 (12.7)
Platinum-based	13 (9.9)	468 (12.6)	481 (12.5)	26 (9.5)	346 (12.0)	372 (11.8)
Non-platinum-based	3 (2.3)	37 (1.0)	40 (1.0)	3 (1.1)	26 (0.9)	29 (0.9)
Other	56 (42.8)	1715 (46.0)	1771 (45.9)	127 (46.5)	1380 (47.7)	1507 (47.6)
Third-line regimen						
Total	101	2808	2909	210	2157	2367
PARPi	4 (4.0)	8 (0.3)	12 (0.4)	4 (1.9)	6 (0.3)	10 (0.4)
Immunotherapy	8 (7.9)	153 (5.5)	161 (5.5)	13 (6.2)	102 (4.7)	115 (4.9)
Other targeted therapy	40 (39.6)	1172 (41.7)	1212 (41.7)	84 (40.0)	873 (40.5)	957 (40.4)
Chemotherapy	49 (48.5)	1475 (52.5)	1524 (52.4)	109 (51.9)	1176 (54.5)	1285 (54.3)
Alkylating agents	8 (7.9)	304 (10.8)	312 (10.7)	15 (7.1)	238 (11.0)	253 (10.7)
Platinum-based	7 (6.9)	290 (10.3)	297 (10.2)	15 (7.1)	227 (10.5)	242 (10.2)
Non-platinum-based	1 (1.0)	14 (0.5)	15 (0.5)	0 (0)	11 (0.5)	11 (0.5)
Other	41 (40.6)	1171 (41.7)	1212 (41.7)	94 (44.8)	938 (43.5)	1032 (43.6)

Data are n (%). BRCA, *BRCA1* and/or *BRCA2***;** BRCAm, BRCA mutation; BRCAwt, BRCA wild-type; HRR, homologous recombination repair; HRRm, homologous recombination repair mutation; HRRwt, homologous recombination repair wild-type; PARPi, poly[ADP ribose] polymerase inhibitor.

**Table A2:** Prevalence of BRCA and HRR mutations by tumor type and disease stage.

	BRCAm			HRRm		
Tumor Type	N	n	Prevalence (95% CI), %	N	n	Prevalence (95% CI), %
Pan-tumor						
Stage I–III	4286	160	3.7 (3.2–4.3)	3209	291	9.1 (8.1–10.1)
Stage IV	2724	82	3.0 (2.4–3.7)	2255	185	8.2 (7.1–9.4)
Bladder cancer						
Stage I–III	455	18	4.0 (2.4–6.2)	317	33	10.4 (7.3–14.3)
Stage IV	232	9	3.9 (1.8–7.2)	185	11	5.9 (3.0–10.4)
Breast cancer						
Stage I–III	887	34	3.8 (2.7–5.3)	665	38	5.7 (4.1–7.8)
Stage IV	242	11	4.5 (2.3–8.0)	190	11	5.8 (2.9–10.1)
Colorectal cancer						
Stage I–III	750	52	6.9 (5.2–9.0)	583	87	14.9 (12.1–18.1)
Stage IV	679	13	1.9 (1.0–3.3)	565	46	8.1 (6.0–10.7)
Non–small-cell lung cancer						
Stage I–III	942	12	1.3 (0.7–2.2)	702	48	6.8 (5.1–9.0)
Stage IV	678	15	2.2 (1.2–3.6)	516	35	6.8 (4.8–9.3)
Pancreatic cancer						
Stage I–III	580	18	3.1 (1.8–4.9)	489	30	6.1 (4.2–8.6)
Stage IV	478	18	3.8 (2.2–5.9)	422	33	7.8 (5.4–10.8)
Prostate cancer						
Stage I–III	672	26	3.9 (2.5–5.6)	453	55	12.1 (9.3–15.5)
Stage IV	415	16	3.9 (2.2–6.2)	377	49	13.0 (9.8–16.8)

BRCAm, *BRCA1* and/or *BRCA2* mutation; HRRm, homologous recombination repair mutation.

**Table A3:** OS analysis using initiation of second-line therapy as index date by HRR biomarker status and tumor type.

Tumor Type	Biomarker Status	N	Median OS (95% CI), mo	Adjusted HR (95% CI)
Bladder cancer	BRCAm	11	17.0 (7.3–NA)	1.56 (0.72–3.37)
	BRCAwt	290	11.7 (9.9–15.5)	
	HRRm	22	14.2 (7.3–NA)	1.10 (0.60–1.99)
	HRRwt	213	11.7 (9.1–16.1)	
Breast cancer	BRCAm	23	22.5 (14.3–NA)	1.02 (0.57–1.84)
	BRCAwt	569	24.6 (20.5–29.0)	
	HRRm	27	19.8 (9.8–42.9)	1.21 (0.71–2.06)
	HRRwt	413	25.5 (22.4–31.5)	
Colorectal cancer	BRCAm	21	25.6 (23.8–NA)	0.62 (0.25–1.52)
	BRCAwt	812	23.7 (21.1–26.3)	
	HRRm	60	33.5 (23.8–NA)	0.58 (0.37–0.90)
	HRRwt	629	22.3 (20.0–26.5)	
Non–small-cell lung cancer	BRCAm	12	19.6 (12.0–NA)	1.54 (0.79–3.02)
	BRCAwt	595	16.3 (14.2–19.1)	
	HRRm	30	12.7 (7.5–23.6)	1.35 (0.86–2.14)
	HRRwt	460	16.6 (14.1–20.1)	
Pancreatic cancer	BRCAm	22	16.0 (4.5–NA)	0.44 (0.25–0.75)
	BRCAwt	552	7.1 (6.4–7.9)	
	HRRm	39	9.0 (7.5–19.8)	0.61 (0.42–0.89)
	HRRwt	463	6.8 (6.0–7.7)	
Prostate cancer	BRCAm	27	38.1 (28.4–NA)	0.91 (0.51–1.61)
	BRCAwt	576	45.2 (38.7– 50.2)	
	HRRm	69	38.7 (28.4–65.0)	0.92 (0.63–1.34)
	HRRwt	478	45.4 (38.6–50.7)	

HRRm, homologous recombination repair mutation; HRRwt, homologous recombination repair wild-type; NA, not available.
